# Cementitious Composites with High Compaction Potential: Modeling and Calibration

**DOI:** 10.3390/ma13214989

**Published:** 2020-11-05

**Authors:** Giao Vu, Tagir Iskhakov, Jithender J. Timothy, Christoph Schulte-Schrepping, Rolf Breitenbücher, Günther Meschke

**Affiliations:** 1 Institute for Structural Mechanics, Ruhr University Bochum, Universitaetsstrasse 150, 44801 Bochum, Germany; thi.vu-h6d@rub.de (G.V.); tagir.iskhakov@rub.de (T.I.); timothy.jithenderjaswant@rub.de (J.J.T.); 2 Institute for Building Materials, Ruhr University Bochum, Universitaetsstrasse 150, 44801 Bochum, Germany; christoph.schulte-schrepping@rub.de (C.S.-S.); rolf.breitenbuecher@rub.de (R.B.)

**Keywords:** compressible cementitious materials, confined compression, Discrete Element Method, deformable grout

## Abstract

There is an increasing need for the development of novel technologies for tunnel construction in difficult geological conditions to protect segmental linings from unexpected large deformations. In the context of mechanized tunneling, one method to increase the damage tolerance of tunnel linings in such conditions is the integration of a compressible two-component grout for the annular gap between the segmental linings and the deformable ground. In this regard, expanded polystyrene (EPS) lightweight concrete/mortar has received increasing interest as a potential “candidate material” for the aforementioned application. In particular, the behavior of the EPS lightweight composites can be customized by modifying their pore structure to accommodate deformations due to specific geological conditions such as squeezing rocks. To this end, novel compressible cementitious EPS-based composite materials with high compaction potential have been developed. Specimens prepared from these composites have been subjected to compressive loads with and without lateral confinement. Based on these experimental data a computational model based on the Discrete Element Method (DEM) has been calibrated and validated. The proposed calibration procedure allows for modeling and prognosis of a wide variety of composite materials with a high compaction potential. The calibration procedure is characterized by the identification of physically quantifiable parameters and the use of phenomenological submodels. Model prognoses show excellent agreement with new experimental measurements that were not incorporated in the calibration procedure.

## 1. Introduction

Difficult geological conditions such as “squeezing rock” [1,2] can induce severe damage to tunnel construction. In general, the time-variant soil deformations increase continuously after completion of the tunnel structure and may severely affect the long term integrity and safety, leading eventually to the complete loss of operability of the tunnel [3]. One possible approach to avoid this substantial risk of damage is the incorporation of lining materials that are highly compressible [4,5,6,7]. These compressible lining materials accommodate large deformations after a certain threshold level of stresses is reached. By allowing the ground to deform, the squeezing pressure acting on the regular concrete tunnel linings will be reduced. Thus, highly compressible lining materials can serve as cushions that protect the regular concrete linings from deterioration. A compressible segmental lining system can be realized either by arranging one or more compressible layers between the lining and the ground (radial compressible systems) or by introducing deformable elements between regular concrete linings (longitudinal/circumferential compressible systems), see in [8] for a discussion on the comparison of various compressible segmental lining systems. Radially compressible systems are in general comprised of a regular rigid cementitious segmental lining, optionally a compressible layer that is attached to this lining during pre-fabrication and a compressible grout that is used to fill the annular gap. A compressible grouting material, in addition to being highly compressible, must satisfy the usual requirements of gap grouting materials. Various compressible materials such as expanded clay [9] to cement based composites such as light weight concrete [10], compressible cementitious mortar with expanded polystyrene [11] or expanded pearls, and foam [12] have been proposed. In general, the basic morphology of a cementitious compressible grout consists of pores and soft inclusions embedded in a cementitious matrix. A laboratory specimen, made of a compressible cementitious composite, that is subject to compressive loads, undergoes three characteristic stages of deformation: (i) an elastic stage with reversible deformations, (ii) a plateau stage where the material undergoes large irreversible deformations, and (iii) a densification stage in which the material has exhausted the compaction capacity and stiffens with increasing loads. At the scale of the microstructure, the material undergoes several complex processes such as reversible topology preserving deformations, gradual collapse of pore/soft-inclusion and compaction of the binder particles.

Designing a compressible grout material with the desired compaction behavior, for a certain specific geological condition, can be challenging as multiple complex phenomena govern the compaction behavior of the material. In order to support the design of novel compressible lining materials and also gain a deeper understanding of the underlying mechanisms, computational simulations can be applied. As these materials are characterized by a highly heterogeneous microstructure with pores and/or soft inclusions [13,14], the type of computational model depends on the desired scale at which the corresponding physical mechanisms are specified: macro-, micro-, or mesoscale. In contrast to macrosopic phenomenological models [15,16,17,18] where the microstructure is treated as a homogeneous medium, continuum micromechanics models take into account the influence of the microstructure using the matrix-inclusion concept [19] in conjunction with inelastic material laws defined at the microscale (elastoplasic [20,21,22] or hyperelastic [23,24,25]) and the utilization of nonlinear homogenization schemes [26,27]. However, as the microstructure is not explicitly resolved but idealized in terms of a representative effective medium, deformation gradients at the scale of microstructure and formation of localization bands cannot be simulated. In contrast, mesoscale models explicitly resolve the microstructure and the interactions between the heterogeneities such as the contact of pore/microcrack faces during compaction. Mesoscale models can be developed using a variety of methods such as the Finite Element Method (FEM) [28,29], the Discrete Element Method (DEM) [30,31], and lattice methods [32,33]. The Discrete Element Method is based on an idealization of the mesostructure using discrete particles, which interact through inter-particle forces and contact mechanisms. This discretization method allows for an explicit representation of the material microstructure with soft [34,35,36] and hard inclusions [37,38,39,40]. Thus, through an explicit representation of the pore structure and by consideration of the pore collapse mechanism, DEM is capable of simulating the essential physics of compressible lining materials. Unfortunately, the calibration of the parameters governing the inter-particle interactions in DEM is generally not easily accomplished directly from laboratory tests, but usually requires inverse identification procedures, see, e.g., in [40,41].

### Goals and Structure of the Paper

In this paper, the behavior of several designs of highly compressible cementitious material composites used to fill the annular gap grouts in mechanized tunneling is investigated using experimental methods. The material behavior of these materials is thereafter analyzed numerically using the Discrete Element Method. Emphasize is laid on the calibration procedure of the inter-particle parameters, which are identified using the data from the experimental procedure. The DEM model is not only intended as a tool for the prognosis of the material behavior, but also to obtain insights into the physical phenomena characterizing the interplay between the changes in the microstructure and the macroscopic deformation behavior.

The remainder of this paper is structured as follows. In Section 2, the theoretical background of DEM and the DEM constitutive contact model for cement paste are reviewed. Section 3 summarizes the experimental data, and discussed the calibration and the validation process. Finally, a discussion of the main findings and concluding remarks are provided in Section 4.

## 2. Modeling Cementitious Materials with DEM

In Discrete Element Method (DEM) models, the material is described as an assembly of particles that can collide, interact, and exert forces on each other. The dynamics of these particles is governed by Newton’s second law. Within this modeling framework, concrete and rock materials are characterized by a packing of particles linked together by cohesive frictional forces. Within the medium, the induced forces are transmitted via a contact network between particles [30].

The contact network is first established and updated by identifying the particles and their nearest neighbor interactions. Thus, particles are considered to interact with each other, if the following condition is fulfilled,

(1) $$\begin{array}{c} l_{ij}\leq R_{I}\left(r_{i}+r_{j}\right), \end{array}$$

where $r_{i}$ and $r_{j}$ are the radii of particles *i* and *j*, and $l_{ij}$ denotes the distance between their centers. $R_{I}$ is the interaction factor set equal to 1 for granular materials. However, for cohesive frictional materials such as concrete, $R_{I}$ is often set to a higher value, e.g., to 1.5 to increase the number of average cohesive contacts per particle, which represents the cohesive properties of the concrete matrix [36,42]. Most DEM formulations are based on the soft-sphere approach, where contact is characterized by an interaction between overlapping particles. However, in the hard-sphere approach [43], contact occurs without allowing an overlap between rigid particles and is instantaneous. An instantaneous point-contact event between rigid spheres in the hard-sphere approach is rather simplistic and this method cannot account for multiple simultaneous contacts between a large number of particles as well as inelastic interactions between particles. A detailed explanation of these two approaches can be found in [44]. In this work, the soft-sphere approach is used.

The interaction forces are evaluated based on the relative displacements in the current particle configuration. Next, the resultant interaction forces are used together with the applied external forces as input for the equations of motion in the time integration step to solve for the new position of all particles.

### 2.1. Governing Equations of Motion

For every particle *i*, the resultant force F${}_{i}$ is the sum of an external force $F_{i}^{ext}$, a damping force $F^{damp}$ and a contact force $F^{ij}$, where *j* defines particles which are in contact with particle *i*:

(2) $$\begin{array}{cc} F_{i} & =F_{i}^{ext}+\sum_{j=1}^{N}F^{ij}+F^{damp}, \end{array}$$

where N is the number of particles having an interaction with particle i.

Numerical damping $F^{damp}$ [36,42] is applied to all particles. The damping term dissipates the overall kinetic energy and ensures quasi-static equilibrium conditions. Given the resultant force F${}_{i}$ on particle *i* having mass *m*, the velocity v${}_{i}$ is evaluated using Newton’s second law as

(3) $$\begin{array}{c} v_{i}^{n+\frac{1}{2}}=v_{i}^{n-\frac{1}{2}}+\frac{F_{i}^{n}}{m_{i}}\Delta t, \end{array}$$

where Δt is the time increment.

Similarly, the effective moment $M_{i}$ and the angular velocity $\omega_{i}$ are updated at each time step:

(4) $$\begin{array}{c} M_{i}=M_{i}^{ext}+\sum_{j=1}^{n_{j}}\frac{M^{ij}}{I_{i}}+M^{damp}, \end{array}$$

(5) $$\begin{array}{c} \omega_{i}^{n+\frac{1}{2}}=\omega_{i}^{n-\frac{1}{2}}+{\dot{\omega }}^{n}\Delta t, \end{array}$$

where $I_{i}$ is the moment of inertia of particle *i*.

The displacement $u_{i}^{n+1}$ and orientation $\theta_{i}^{n+1}$ are updated according to

(6) $$\begin{array}{cc} u_{i}^{n+1} & =u^{n}+v_{i}^{n+\frac{1}{2}}\Delta t, \end{array}$$

(7) $$\begin{array}{cc} \theta_{i}^{n+1} & =\theta_{i}^{n}+\omega_{i}^{n+\frac{1}{2}}\Delta t. \end{array}$$

As a result, the position of all particles is updated accordingly. In the next time step, the newly updated configuration is used to resolve the interaction between particles in terms of contact stresses and strains.

### 2.2. Constitutive Law

Given the updated position of all particles, the interaction forces in the contact network are computed. The normal and tangential strains $\varepsilon_{n},\varepsilon_{\tau }$ are computed based on the relative normal and tangential displacements $u_{n},u_{\tau }$ between two particles and their initial distance $l_{0}^{ij}$:

(8) $$\varepsilon_{n}=\frac{u_{n}}{l_{0}^{ij}},\;\varepsilon_{\tau }=\frac{u_{\tau }}{l_{0}^{ij}}.$$

The normal relative displacement vector is calculated as

(9) $$u_{n}=(∥x_{i}-x_{j}∥-l_{0}^{ij})n,$$

where $x_{i},x_{j}$ denote the current positions of the particle centroids and n is the normal vector connecting the centroids of two particles. The relative tangential displacement is computed by subtracting the normal component from the total relative displacement $u^{ij}$

(10) $$u_{\tau }^{ij}=u^{ij}-\left(u^{ij}\cdot n\right)n,$$

where

(11) $$u^{ij}=\left(\Delta u_{i}-\Delta u_{j}\right)+\left(\omega_{i}\times r_{ci}-\omega_{j}\times r_{cj}\right)\Delta t.$$

$\Delta u^{i}$ and $\Delta u^{j}$ are the displacement increments of particles *i* and *j*, and $r_{ci}$ and $r_{cj}$ denote the vectors connecting the point of the contact and centroid of particles.

The elastic behavior between two particles is characterized by the normal $K_{n}$ and tangential $K_{\tau }$ stiffness moduli. The normal stress and shear stress are computed directly from the updated position as

(12) $$\sigma_{n}=K_{n}\varepsilon_{n},\;\sigma_{\tau }=K_{\tau }\varepsilon_{\tau }.$$

**Figure 1:**
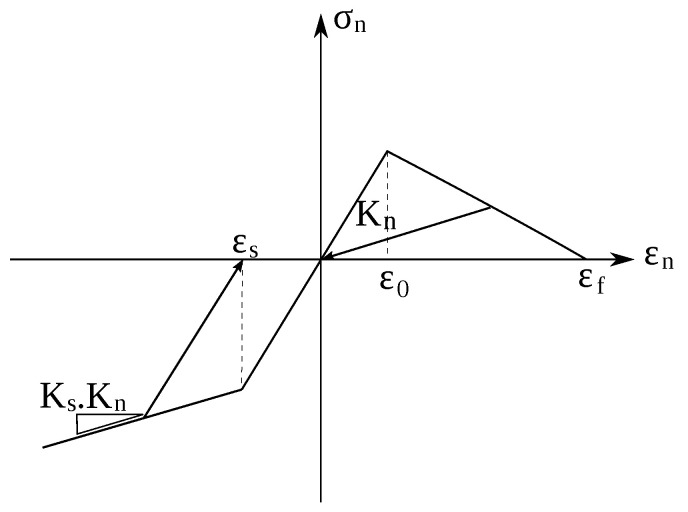
Contact behavior between two particles subjected to tension in normal direction.

In this work, the concrete contact modeling approach according to the work in [45] was adopted, where the interaction of the particles tension in normal direction is governed by a damage softening law

(13) $$\begin{array}{c} \sigma_{n}=\left[1-\omega H\left(\varepsilon_{n}^{e}\right)\right]K_{n}\varepsilon_{n}^{e}. \end{array}$$

where ω is a damage parameter (ω∈[0,1]) and $H(\epsilon_{n})$ is the Heaviside function, used to deactivate damage in compression. The linear softening law is characterized by the predefined limit elastic strain $\varepsilon_{0}$ and the ultimate strain $\varepsilon_{f}$, as shown in Figure 1.

The normal compression mode is characterized by an elasto-plastic behavior with plastic strain $\varepsilon_{s}$ and the relative hardening stiffness $K_{s}$ (Figure 1)

(14) $$\begin{array}{cc} \varepsilon_{n} & =\left\{\begin{array}{cc} \varepsilon_{n}+\frac{\sigma_{n}}{K_{n}}, & \mathrm{if}\;\sigma_{n}<\sigma_{s},\\ \varepsilon_{s}+\frac{\sigma_{n}-K_{n}\varepsilon_{s}}{K_{s}K_{n}}, & otherwise. \end{array}\right. \end{array}$$

**Figure 2:**
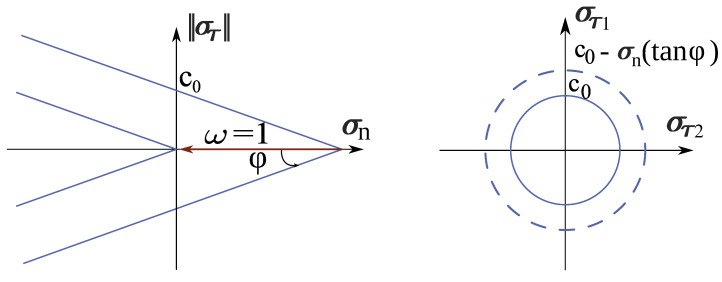
Illustration of the Mohr–Coulomb yield surface. As the damage parameter ω increases, the yield surface is shifted along the normal stress axis in negative direction.

Shear stress can be calculated from the modified Mohr–Coulomb frictional law $f(\sigma_{n},\sigma_{\tau })$, taking into account the damage parameter ω (see Figure 2):

(15) $$\begin{array}{c} f\left(\sigma_{n},\sigma_{\tau }\right)=\left|\right|\sigma_{\tau }\left|\right|-\left[c_{0}\left(1-\omega \right)-\sigma_{n}\tan\varphi \right], \end{array}$$

where $c_{0}$ is the initial cohesion and φ is the frictional angle. Shear stress and strain are updated as follows. First, the trial shear stress is computed

(16) $$\begin{array}{cc} \sigma_{\tau }^{trial} & =K_{\tau }\varepsilon_{\tau }, \end{array}$$

then the shear stress is corrected according to

(17) $$\begin{array}{cc} \sigma_{\tau } & =\left(c_{0}\left(1-\omega \right)-\sigma_{n}\tan\varphi \right)\frac{\sigma_{\tau }^{trial}}{|\sigma_{\tau }^{trial}|}. \end{array}$$

Finally, the shear strain is recomputed as

(18) $$\begin{array}{cc} \varepsilon_{\tau } & =\frac{\sigma_{\tau }}{|\sigma_{\tau }^{trial}|}\frac{\varepsilon_{\tau }}{|\varepsilon_{\tau }|}. \end{array}$$

Given the contact stresses, the contact forces are obtained as

(19) $$\begin{array}{cc} F_{n}^{ij} & =\sigma_{n}^{ij}A^{ij}n, \end{array}$$

(20) $$\begin{array}{cc} F_{\tau }^{ij} & =\sigma_{\tau }^{ij}A^{ij}, \end{array}$$

where $A^{ij}$ is the contact interface area [36] which is defined as $A^{ij}=\pi min{\left(r^{i},r^{j}\right)}^{2}$.

All parameters of the described constitutive model summarized in Table 1 are to be calibrated according to the properties of the specific material to be analyzed. The calibration procedure for cementitious compressible composites is given in the following Section.

## 3. Calibration and Validation of the DEM Model Based on Laboratory Experiments

### 3.1. Experimental Data from Compression Tests on Highly Compressible Composite Grouts

**Figure 3:**
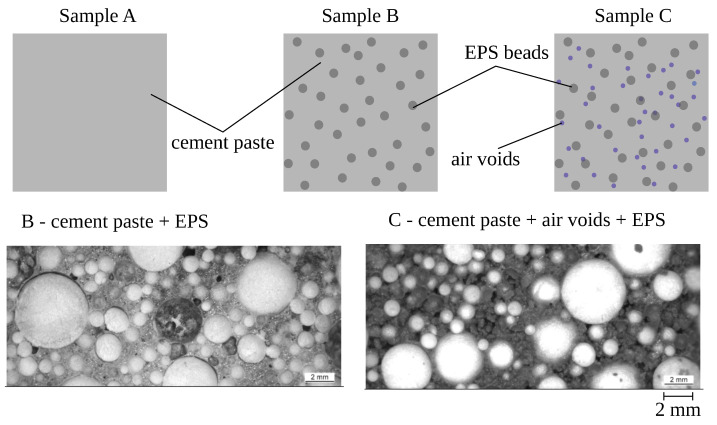
(**Top**): Schematic illustration of three different grout mixes prepared within the experimental program. (**Bottom**): Direct light microscope images of hardened grout samples B and C.

**Figure 4:**
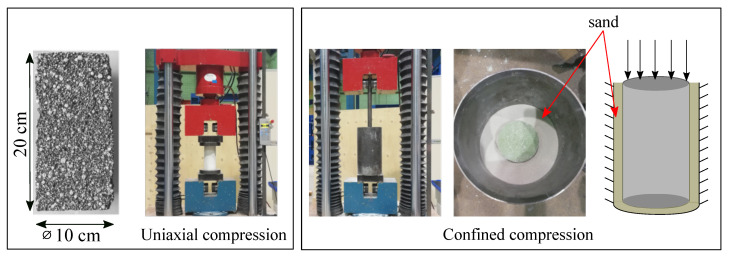
(**Left**): Sample geometry and experimental set-up for the uniaxial compression test. (**Right**): Experimental setup for the confined compression test.

As possible candidates for annular gap grout, three different activated two component-grout mixes with a defined fraction of expanded polystyrene (EPS) beads (denoted as A, B, and C) have been prepared within the experimental program. The type of binder is the same for all mixes. Mix A consists of only cement binder matrix (porosity < 1%); mix B consists of cement paste and EPS beads with a volume fraction of 61.5%; and mix C consists of cement paste, EPS, and additional air voids, contributing up to 71.1% of volume fraction in total (see Figure 3 and Table 2). Two cylindrical samples with diameter of 100 mm and height of 200 mm (see Figure 4 left) of each mix have been casted and cured for 7 days.

The samples were subjected to both unconfined uniaxial and confined uniaxial compression tests with a constant loading rate of 20 mm/min (or strain rate $\dot{\varepsilon }=0.1$ s^−1^). First, a uniaxial compression test (see Figure 4 left) was performed on sample A to obtain the mechanical properties of the cement paste. Then, uniaxial compression and confined compression (see Figure 4 right) tests were conducted on samples B and C to investigate the effect of EPS and air voids on the mechanical behavior of the grout. To simulate confinement, the sample was placed in a steel container (diameter = 180 mm) filled with fine sand (see Figure 4 right) in the confined tests. All tests were performed using displacement control.

**Figure 5:**
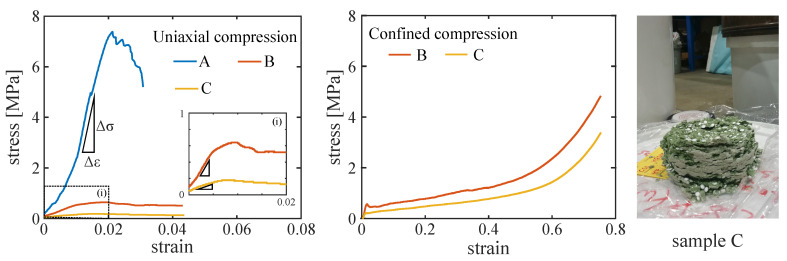
(**Left**): Experimentally measured response of the samples A, B, and C under uniaxial compression. (**Middle**): Experimentally measured response of composites B and C under confined compression. (**Right**): Sample C at the final state of compaction.

Table 3 presents elasticity modulus, compressive strength and density of samples A, B, and C as the outcome of the uniaxial compression tests (Figure 5 left). Young’s moduli of samples A, B, and C are estimated using the stress and strain intervals indicated by black lines in Figure 5 left. Grout samples B and C, owing to the high void content, exhibited a decrease in strength and stiffness as compared to sample A. In contrast to sample A, which failed due to tensile splitting, in samples B and C damage initiated at the upper part where the load was applied, followed by local crushing.

In the confined compression test, the behavior of samples B and C differs from sample A. Three distinct stages of deformation were observed in samples B and C under confined compression: An elastic region, a plateau and a densification stage (Figure 5 middle). After the elastic stage, in the plateau stage, the material undergoes plastic deformation characterized by large plastic deformations with marginal increase in stresses, associated with the collapse of voids. When the pores have completely collapsed, subsequent to the plateau stage, densification due to pore compaction results in a significant increase in the tangent stiffness of the material. The confinement condition has prevented the samples from failure resulting from lateral expansion. In the confined compression tests, irreversible compaction of the composites up to 75% was attained (Figure 5 right)).

### 3.2. Calibration of Model Parameters

Using the data obtained from the experiments, we proceed to calibrate the model parameters as follows. (a) Data obtained from uniaxial compression of sample A is used to calibrate the contact parameters for the cement paste matrix; (b) data obtained from uniaxial compression tests on samples B and C is used to calibrate the porosity of DEM numerical models for the samples B and C; (c) the stress–strain curve of B under confined compression is further used for calibrating additional DEM parameters associated with the compression behavior of the material; (d) finally, the data from the confined compression experiments using sample C is exclusively used for the validation of the model.

**Figure 6:**
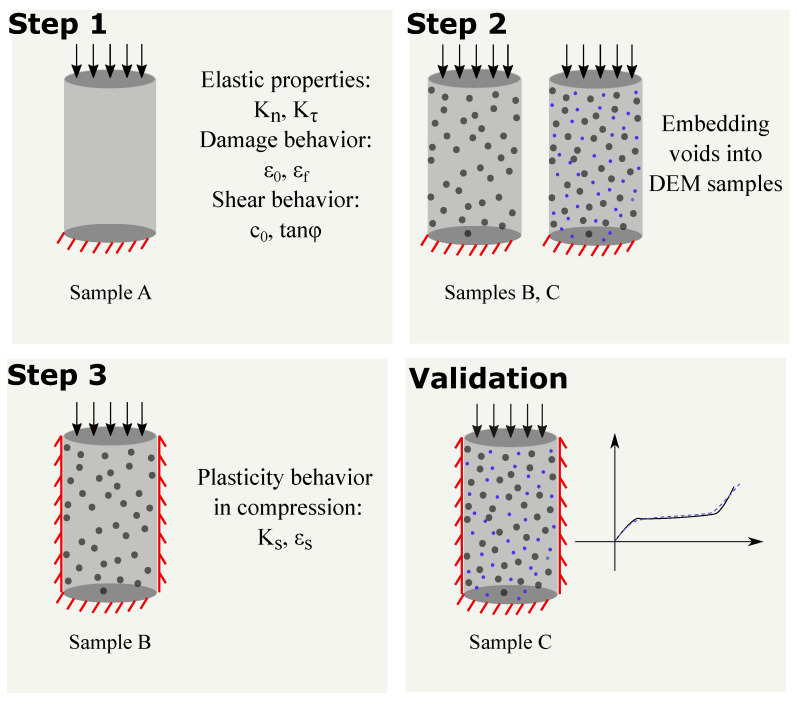
Calibration procedure. Step 1: Calibration of contact parameters using the uniaxial response of sample A. Step 2: Generating composite samples by embedding spherical voids representing the air voids and the expanded polystyrene (EPS) inclusions into the numerical DEM model for sample A. The voids are embedded into the DEM specimen by removing the DEM particles lying within the spherical region defined by the void location. Step 3: Calibration of plasticity parameters of the DEM model to match the compaction behavior of sample B under confined compression. Validation: The stress–strain response obtained by the DEM simulation of sample C under confined conditions is compared with laboratory results for sample C.

The calibration of parameters for the described model is performed following three steps according to the procedure illustrated in Figure 6.

#### 3.2.1. Calibration Step 1

First, a computational model of a cylindrical sample of height 200 mm and diameter 100 mm has been generated for the DEM simulation as shown in Figure 8 left. In DEM, a sample is an assembly of spherical particles occupying a given geometry and is often referred to as packing. Here, a dense packing with a random arrangement was chosen. DEM particles of the same size (radius = 0.8 mm) were used. This packing is assumed to represent the cement paste matrix without EPS or air voids (sample A), which results in a total of 452,257 particles. However, these DEM particles do not represent the actual morphology of the cement paste, rather being a means of material discretization at the mesoscale. In order to resolve the actual geometrical morphology of the cement paste, including the complete pore space ranging from a few nanometers up to a few micrometers, would require a tremendous amount of computational power. For cementitious materials, the interaction factor $R_{I}$ is chosen to be 1.5 [42,46].

Next, the uniaxial compression simulation was performed with this numerical sample analogous to the experimental test. In the model, particles at the bottom cylinder face were fixed in all directions while at the top face a vertical constant strain rate was applied. In order to simulate quasi-static conditions, numerical damping with a damping factor of 0.3 was adopted [45,47] to dissipate the total kinetic energy of the system. Moreover, mass scaling of (4800 kg/m^−3^) was adopted to increase the critical time step [45,47], which is a standard value for modeling concrete using the DEM. This allows to apply strain rates that are is not too small to enable reasonable computational costs of the analyses. Furthermore, a constant loading velocity of $5\times 10^{-2}$ m/s was set in all simulations to exclude the effect of inertia. During the simulations, the resultant forces and displacements at the top cylinder face were recorded and used to compute the stress and the deformation.

**Figure 7:**
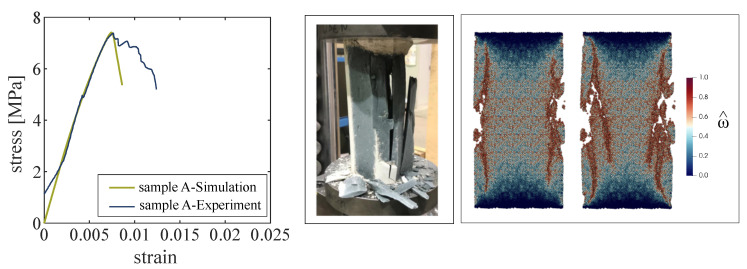
(**Left**): Uniaxial compression of sample A: Load–displacement curves obtained from the experiment (black line) and the DEM simulation (green line). (**Middle**): Photo of the damaged sample A after the uniaxial compression test in the laboratory. (**Right**): Damage pattern obtained from the DEM simulation at two stages of compressive loading: at 6.714 kN/cm^2^ (post peak), and 5.461 kN/cm^2^ (post peak) in the cross section of the cylindrical sample A.

As a result, by matching the experimentally measured elastic properties and the compresssive strength of sample A (see Figure 7 left) the normal and tangential elasticity moduli ($K_{n}$, $K_{\tau }$), the parameters of the damage law ($\varepsilon_{0}$, $\varepsilon_{f}$) and the Mohr–Coulomb yield surface parameters ($c_{0}$, tanφ) are calibrated (Table 4). It is noted that the early portion of the load displacement curve obtained from the experiments in Figure 7 left shows a nonlinear behavior, which is attributed to the loading discrepancy between the specimen and loading plate in the initial stage of loading, a phenomenon commonly observed in laboratory testing [48]. This initial disturbance has been filtered out and does not affect the calibration procedure.

To visualize the damage pattern, we define the parameter ${\hat{\omega }}^{i}$, which is a damage parameter averaged over all cohesive contacts (i.e., contacts that are created at the initiation step) associated with particle *i*. Accordingly, ${\hat{\omega }}^{i}$ of particle *i* is defined as

(21) $$\begin{array}{c} {\hat{\omega }}^{i}=\frac{\sum_{j=1}^{n_{j}}\omega^{ij}}{n_{j}}, \end{array}$$

where $\omega^{ij}$ is the damage parameter of the normal interaction between particle *i* and particle *j*, and $n_{j}$ is the number of initial cohesive bonds of particle *i*. Particles with ${\hat{\omega }}^{i}=0$ are in an undamaged state, while ${\hat{\omega }}^{i}=1$ indicates a fully damaged state.

Figure 7 illustrates the damage pattern in sample A in the experiment (middle) and in two stages of uniaxial compression in the numerical simulation (right). The figures show a cross section through the cylindrical sample. In the simulation, damage first occurs by the formation of a macrocrack near the outer surface, almost parallel to the loading axis, followed by a secondary, inclined macrocrack. The photo from the damaged specimen after the end of the test also shows dominant vertical cracks at both edges of the specimen, partially with a slight inclination as was observed in the results from the DEM simulations.

#### 3.2.2. Calibration Step 2

Ideally, the microstructure of the material used in the experiment should serve as the direct input to generate the numerical sample for DEM simulation, e.g., by incorporating data from CT imaging techniques in DEM models with a realistic microstructure (i.e., cement paste, aggregates, ITZ, etc.) [38]. However, in this paper, the composite microstructure of the investigated grout mixes is characterized by inclusions (pores, air voids, and EPS beads) at multiple scales. Thus, it would require a tremendous amount of very small DEM particles to resolve the topology of the small inclusions. This would be computationally too expensive. Therefore, the numerical models for the samples B and C have been created without CT scans by “embedding” spherical voids into the numerical DEM model for sample A generated and calibrated in Step 1. The voids are embedded into the DEM specimen by removing the DEM particles lying within the spherical region defined by the void location. These spherical voids represent both air voids and EPS beads, as the stiffness of EPS is comparatively low. Thus, from modeling perspective we do not distinguish between air voids and EPS beads.

Using the DEM model with embedded voids, a uniaxial compression test was simulated (same as in Step 1), and the compressive strength in uniaxial test was recorded. Voids were embedded in an iterative manner until the compressive strength of the sample obtained in simulation matches the experimentally measured compressive strength of the corresponding mixes B and C. It must be noted that, DEM particles do not represent the actual cement paste grains, and that at this level of observation, they are rather a means to discretize the material at the mesoscale. Consequently, certain parameters controlling, e.g., plasticity in shear and in normal compression, have to be employed to capture the correct physics of interactions at the lower scale.

The process of embedding the voids into the DEM sample is as follows.

- Voids with a prescribed volume fraction and size distribution are randomly picked and placed within a cylindrical domain (height = 200 mm, diameter = 100 mm).
- The coordinate and radius of each void particle is recorded.
- Given a dense packing of DEM particles generated in Step 1, the DEM particles lying within the spherical region defined by void position and radius are removed.

**Figure 8:**
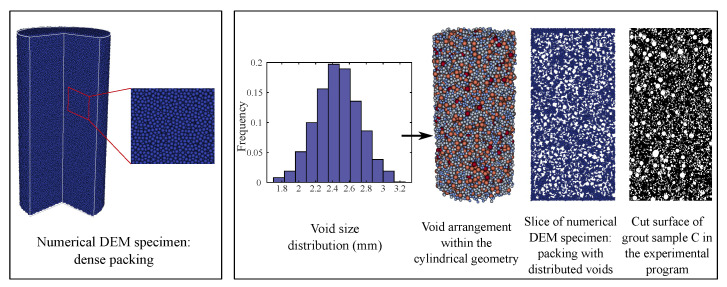
(**Left**): Numerical model of an initial sample A with a dense packing constituting the basis for the generation of DEM models B and C with air voids. (**Right**): Generation of numerical DEM model for samples with distributed voids and comparison with a photo from the cut surface of sample C.

The void size distribution was assumed to follow the normal probability density function with a mean radius of 2.5 mm and standard deviation of 0.25 (see Figure 8 right). The numerical packing based on this void size distribution can be qualitatively compared with the void distribution along the cut surface of the grout sample prepared in the experimental program (see Figure 8 right).

The geometrical data representing the air voids embedded in the numerical models for samples B and C are summarized in Table 5.

**Figure 9:**
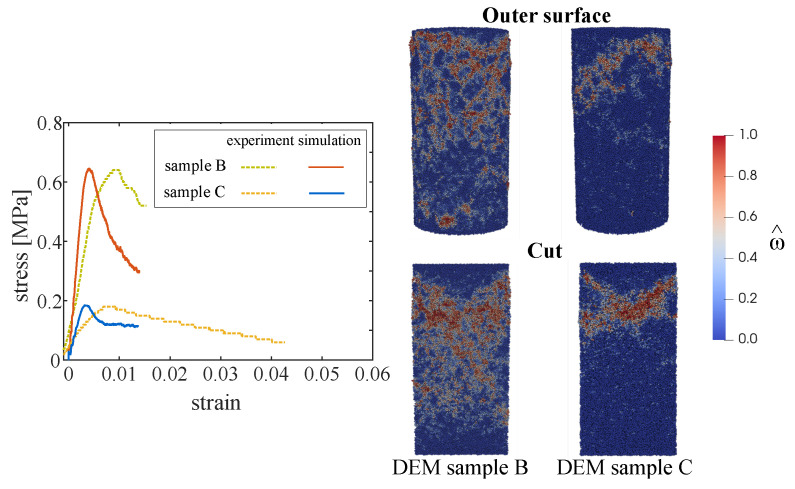
(**Left**): Uniaxial compression of samples B and C: comparison of experimental measurements and numerical results. (**Right**): Distribution of the damage parameter $\hat{\omega }$ obtained from the DEM simulation at strain level 0.008 for sample B (void volume fraction = 42%) and sample C (53.2%).

Figure 9 right shows the distribution of fracture, represented by the damage parameter $\hat{\omega }$, according to the DEM simulations of the samples corresponding to mixes B and C at at strain level 0.008. $\hat{\omega }$ is the damage parameter averaged over all contacts of a corresponding particle. At the top, the damage distribution at the outer surface is illustrated, and at the bottom, the damage distribution along the cross section of the specimen is illustrated.

In comparison to the experimental data, uniaxial compression simulations on the generated samples with voids yielded higher stiffness for the same strength level (see Figure 9 left). This is due to the relatively coarse discretization which does not capture small voids present in the real material. Initially, prior to reaching the peak stress, microcracking is predicted to initiate diffusively at different locations in the specimen, mostly localized in the vicinity of voids. As the load is increased, damage starts to localize at the upper part of the specimen, followed by local crushing/compaction (see Figure 9 right, which shows the state of damage at the axial strain level ε=0.008 in the post-peak state).

#### 3.2.3. Calibration Step 3

**Figure 10:**
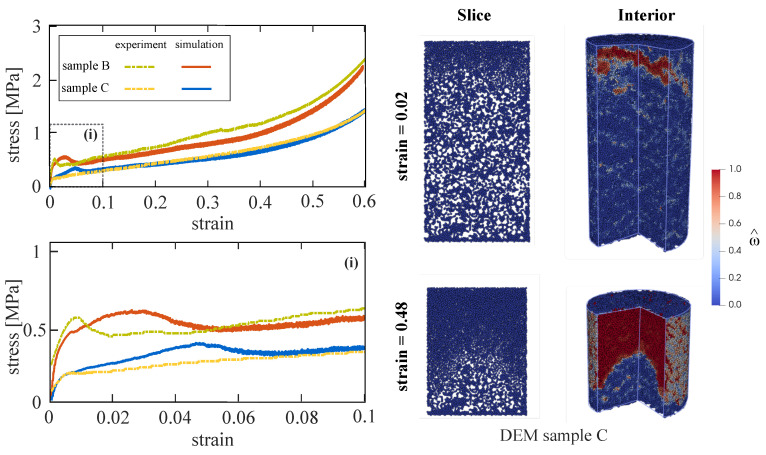
(**Left**): Calibration step 3 and model validation: Results from DEM simulations of confined compression tests on samples B and C and comparisons with test results. The experimentally observed behavior of mix B (green dotted line) was used for the calibration of the model plasticity parameters (red lines). The numerical results for sample C (blue lines) are predictions and must be validated against the experimental results (yellow dotted lines). (**Right**): Distribution of damage in the cross section and interior of specimen predicted by the DEM model for sample C under confined compression in two stages of compressive loading.

In the previous two calibration steps, the numerical DEM models for samples representing mixes B and C have been generated and calibrated based on the uniaxial compression experiments performed with mixes B and C. In Step 3, the simulation of confined compression on numerical sample B is performed and compared to experimental data in order to calibrate the plasticity parameters $K_{s}$, $\varepsilon_{s}$ (Table 4). Figure 10 left shows the comparison of the stress-strain response for mix B obtained from the calibrated DEM model (red lines) and in the laboratory test (green dotted lines).

### 3.3. Validation

In the calibration procedure, the normal and tangential elasticity moduli ($K_{n}$, $K_{\tau }$), the softening curve parameters ($\varepsilon_{0}$, $\varepsilon_{f}$) and parameters defining the Mohr–Coulomb yield surface ($c_{0}$, tanφ) have been calibrated in Step 1 based on the uniaxial compression experiments with mix A. In Step 2, the microstructure topology of the composites corresponding to mixes B and C has been characterized by matching the compressive strength obtained numerically to the measured data using uniaxial compression tests on samples B and C. In Step 3, the plasticity parameters $K_{s}$, $\varepsilon_{s}$ have been calibrated based on the experimentally observed material response of sample B in the confined compression test.

After calibrating the required parameters, the model is now used to predict the complete behavior of sample C under confined compression until a strain level of 0.6. The uniaxial unconfined compression test was used to calibrate the volume fraction of embedded voids for sample C. The experimental results from sample C under confined compression were not used to calibrate any model parameter. This ensures the predictive capability of the model. Figure 10 left presents the predicted (blue lines) and experimentally observed stress–strain response (yellow lines) for the sample C under confined compression conditions.

In the DEM simulation, initially diffuse cracking occurs arbitrarily within the whole specimen. This plateau stage is characterized by material compaction and a slight stress increase. According to the computational model, damage initiates at the top part of the sample and forms a compression band which propagates downwards during the pore compaction process. This leads to a material compaction gradient which can be clearly seen in Figure 10 right top. 

During the pore compaction process, most pores collapse “layer by layer”. Particles with all contacts “broken” dynamically interact with each other by means of newly created interactions (i.e., interaction between two particles as they “collide” with each other) and rearrange themselves to fill the voids (Figure 10 right bottom). This mechanism is reflected by a high material compaction without significant change in stress leading to the plateau behavior of the stress–strain curve also observed in the experiments. As soon as all voids have experienced collapse, a densification process initiates, which is characterized by the regain in stiffness. Moreover, this is in agreement with the observations in the laboratory.

In this work, all simulations were performed using an open-source DEM software ‘WooDEM’ [49]. The software is written in C++11 and supports OpenMP parallelization. The computational time required for the simulation of the confined compression test on sample B (Figure 10 left) is 207 h for $2.4\times 10^{6}$ time steps. Each simulation is performed on 12 Intel^®^ Xeon^®^ Gold 6148 CPUs running in parallel @ 2.40 GHz with 100 GB RAM.

## 4. Conclusions

In this paper, we have generated a computational model employing the Discrete Element Model to simulate the behavior of specimens made of highly compressible cementitious materials. To enable a systematic calibration procedure, two different concrete mixes as well as a specimen made of cement paste only have been tested experimentally. One of the mixes contained EPS beads of different sizes, and the second mix also contained, in addition, artificial air voids. Cylindrical samples based on selected material designs have been subjected to compressive loads with and without lateral confinement. The calibration procedure used information from the test on cement paste specimens as well as the compressive strength data from the highly compressible specimens with only EPS beads subjected to uniaxial compression. The DEM model was eventually validated based on test results from a highly compressible specimen with a material mix also containing air voids subjected to confined compression. Using this calibration procedure, the main physical mechanisms associated with compaction processes occurring in the specimens could be well captured by means of the DEM model. The model has shown a cascade-type mechanism of pore collapse, which propagates from the top to the bottom. This mechanism was also observed in the experiments. After the compaction process was completed, the model showed, again in agreement with the experiments, a re-stiffening of the material. From the computational and experimental analysis, the following conclusions are drawn.

- Cementitious materials with high-compaction potential can be designed using a combination of weak inclusions and pores.
- Experimental observations and model simulations show the development of compaction gradients during confined uniaxial compression tests.
- Despite the extensive work dedicated to the calibration procedure as well as the high computational cost, DEM has shown its capability to replicate the main physical mechanisms governing the behavior of compressible cementitious composites.
- In order to capture the effect of fine pores with a characteristic size smaller than the DEM discretization, a phenomenological plasticity-type submodel has to be calibrated in addition to the usual inter-particle parameters.
- The proposed calibration procedure offers a good control of the pore structure characteristics, such as void volume fraction, air-void size, and void size distribution. Consequently, the proposed computational model allows to support the design of new materials with specific, customized compaction properties (elastic phase, plateau, and densification). These materials can be used for optimizing the compressibility characteristics of annular gap grouts used to fill the tail void gap in mechanized tunneling in case of tunneling projects in rocks with a high squeezing potential.

As an outlook, other materials for inclusions enabling a controlled compaction behavior of cementitious materials will be considered, which are characterized by a crushing mechanism when subjected to large compressive stresses. One candidate for such a damage tolerant composite material is based on using expanded glass beads as inclusion. This concept is currently explored in laboratory tests.

## Figures and Tables

**Table 1:** Summary of the model parameters required in calibration of the Discrete Element Method (DEM) model.

Elastic parameters		
$K_{n}$	normal modulus	Pa
$K_{\tau }$	tangential modulus	Pa
**Damage law in tension**		
$\varepsilon_{0}$	limit elastic strain	
$\frac{\varepsilon_{f}}{\varepsilon_{0}}$	relative ductility	
**Elasto-plasticity in shear**		
$c_{0}$	initial cohesion	Pa
tanφ	frictional angle	
**Elasto-plasticity in compression**		
$\varepsilon_{s}$	plastic strain	
$K_{s}$	relative hardening modulus	

**Table 2:** Mixture design for grout samples A, B, and C.

Mix Designs	A	B	C
**Source Materials**	**Volume** [l]	**Volume** [l]	**Volume** [l]
Cement	67.6	25.9	19.4
Slag	142.0	54.5	40.8
Filler	0.0	0.0	0.0
Bentonite	21.4	8.2	6.2
Water	695.9	267.1	200.0
Foaming agent	0.0	0.0	0.89
EPS 0.5–1 mm	0.0	297.9	223.1
EPS 1–2 mm	0.0	123.3	92.3
EPS 2–5 mm	0.0	195.2	146.2
Activator 1 (Sodium)	37.6	14.4	10.8
Activator 2 (Potassium)	35.5	13.6	10.2
Calculated Air voids	0.0	0.0	250.0
Total	1000	1000	1000

**Table 3:** Material properties of grout samples A, B, and C. Young’s modulus and compressive strength are obtained from the uniaxial compression tests (Figure 5 left).

Sample	Young’s Modulus (GPa)	Compressive Strength (MPa)	Density (kg/m^3^)
A	1.262	7.38	1430
B	0.248	0.64	840
C	0.12	0.18	460

**Table 4:** Calibrated parameters of the DEM model for the cementitious matrix.

Elastic parameters		
$K_{n}$	0.8	GPa
$K_{\tau }$	0.2	
**Damage law in tension**		
$\varepsilon_{0}$	$5.5\times 10^{-4}$	
$\frac{\varepsilon_{f}}{\varepsilon_{0}}$	30	
**Elasto-plasticity in shear**		
$c_{0}$	0.25	MPa
tanφ	0.577	
**Elasto-plasticity in compression**		
$\varepsilon_{s}$	$-1.2\times 10^{-3}$	
$K_{s}$	0.001	

**Table 5:** DEM models for samples B and C: Number and size of DEM particles and representation of the air pores.

Sample	Radius of DEM Particles (mm)	Number of DEM Particles	Void Volume Fraction	Mean Void Radius (mm)	Number of Voids
B	0.8	253,718	42%	2.5	11,567
C	0.8	199,357	53.2%	2.5	14,651

## Abbreviations

The following abbreviations are used in this manuscript.

EPS	Expanded polysterene
REV	Representative elementary volume
FEM	Finite Element Method
DEM	Discrete Element Method
